# Protein oxidation and degradation caused by particulate matter

**DOI:** 10.1038/srep33727

**Published:** 2016-09-20

**Authors:** Ching-Huang Lai, Chun-Nin Lee, Kuan-Jen Bai, You-Lan Yang, Kai-Jen Chuang, Sheng-Ming Wu, Hsiao-Chi Chuang

**Affiliations:** 1 School of Public Health, National Defense Medical Center, Taipei, Taiwan; 2 School of Respiratory Therapy, College of Medicine, Taipei Medical University, Taipei, Taiwan; 3 Division of Pulmonary Medicine, Department of Internal Medicine, Shuang Ho Hospital, Taipei Medical University, New Taipei City, Taiwan; 4 Division of Pulmonary Medicine, Department of Internal Medicine, Wan Fang Hospital, Taipei Medical University, Taipei, Taiwan; 5 Department of Public Health, School of Medicine, College of Medicine, Taipei Medical University, Taipei, Taiwan; 6 School of Public Health, College of Public Health and Nutrition, Taipei Medical University, Taipei, Taiwan; 7 Department of Internal Medicine, School of Medicine, College of Medicine, Taipei Medical University, Taipei, Taiwan

## Abstract

Particulate matter (PM) modulates the expression of autophagy; however, the role of selective autophagy by PM remains unclear. The objective of this study was to determine the underlying mechanisms in protein oxidation and degradation caused by PM. Human epithelial A549 cells were exposed to diesel exhaust particles (DEPs), urban dust (UD), and carbon black (CB; control particles). Cell survival and proliferation were significantly reduced by DEPs and UD in A549 cells. First, benzo(a)pyrene diolepoxide (BPDE) protein adduct was caused by DEPs at 150 μg/ml. Methionine oxidation (MetO) of human albumin proteins was induced by DEPs, UD, and CB; however, the protein repair mechanism that converts MetO back to methionine by methionine sulfoxide reductases A (MSRA) and B3 (MSRB3) was activated by DEPs and inhibited by UD, suggesting that oxidized protein was accumulating in cells. As to the degradation of oxidized proteins, proteasome and autophagy activation was induced by CB with ubiquitin accumulation, whereas proteasome and autophagy activation was induced by DEPs without ubiquitin accumulation. The results suggest that CB-induced protein degradation may be via an ubiquitin-dependent autophagy pathway, whereas DEP-induced protein degradation may be via an ubiquitin-independent autophagy pathway. A distinct proteotoxic effect may depend on the physicochemistry of PM.

Air pollution consists of numerous reactive oxygen species (ROS), which are associated with the development of cardiopulmonary disease^1^. Particulate matter (PM), in particular, is capable of directly producing ROS due to its unique physicochemistry (e.g., organic and inorganic compounds). Dysfunction of mitochondria or NADPH-oxidase and activation of inflammatory cells to produce ROS and reactive nitrogen species (RNS) are also caused by PM^2^,^3^. Although extracellular ROS can be mitigated or delayed by antioxidants in biological systems, an overload of ROS is able to attack local tissues leading to cell injury (e.g., mitochondrial and DNA damage) and consequently resulting in cell death (e.g., necrosis and apoptosis)^4^,^5^.

Notably, ROS can damage surrounding tissues and result in the formation of highly reactive organic molecules, which are able to non-enzymatically modify proteins and that target specific peptide residues^6^. Sulfur-containing residues, such as methionine and cysteine, are responsible for more than 70% of ROS interactions with proteins^7^,^8^. Pardo and colleagues showed that repeated exposure to urban PM caused protein oxidation in mouse lungs^9^. Those authors suggested that chemical compounds may be important in regulating protein oxidation. Our previous study further showed that methionine plays an important role in response to ambient fine particle exposure in the lungs of mice^10^. ROS oxidize methionine to methionine sulfoxide (MetO; oxidized methionine) which forms two enantiomers: *S*-MetO and *R*-MetO. The peptide MetO enzymes, methionine sulfoxide reductase A (MSRA) and MSRB, are able to catalytically reverse *S*-MetO and *R*-MetO, respectively, allowing maintenance of the protein’s function in some cases^11^.

Approximately 90% of damaged proteins are degraded into small peptides by the ubiquitin-proteasome pathway^12^, which plays a critical role in removing oxidized proteins and maintaining biological systems in a healthy status. The ubiquitin-proteasome pathway is modulated by the cellular redox status^13^. For example, mild or transient ROS transiently increase the ubiquitination system and proteasome activity and enhance proteolysis. Severe or persistent levels of ROS cause dysfunction of the ubiquitin-proteasome pathway and inactivate proteolysis activity. Human studies showed that acute exposure to diesel exhaust and secondary organic aerosols reduced proteasome activity in peripheral blood cells^14^,^15^, suggesting that proteasomes are an important mechanism in response to acute exposure of air pollution.

Beside the ubiquitin-proteasome pathway, autophagy is also an essential pathway to degrade oxidized proteins. Autophagy is a regulated cellular mechanism for degrading proteins that is mediated by lysosomal-dependent processing. The autophagosome then fuses with and delivers its contents to the lysosome. Lysosomal enzymes subsequently facilitate degradation to regenerate metabolic precursor molecules. Increasing numbers of studies have demonstrated that autophagy expression is associated with air pollution^16^,^17^. Recently, ubiquitination functions were found to play a general role in selective autophagy in mammalian cells. Autophagy receptors, such as p62 and NBR1, that simultaneously bind both monoubiquitinated and polyubiquitinated substrates act as adaptors between ubiquitination and autophagy^18^. However, little is known about the effects of ambient PM on selective autophagy.

The objectives of this study were: (1) to discover the potency of protein oxidation caused by different types of particulate air pollution; (2) to investigate the MSR repair system for methionine oxidation caused by particles; (3) to understand the regulation of ubiquitin-proteasome pathways after particle exposure; and (4) to examine selective autophagy mediated by particles.

## Results

### Characterization of CB, DEPs, and UD

**Figure 1:**
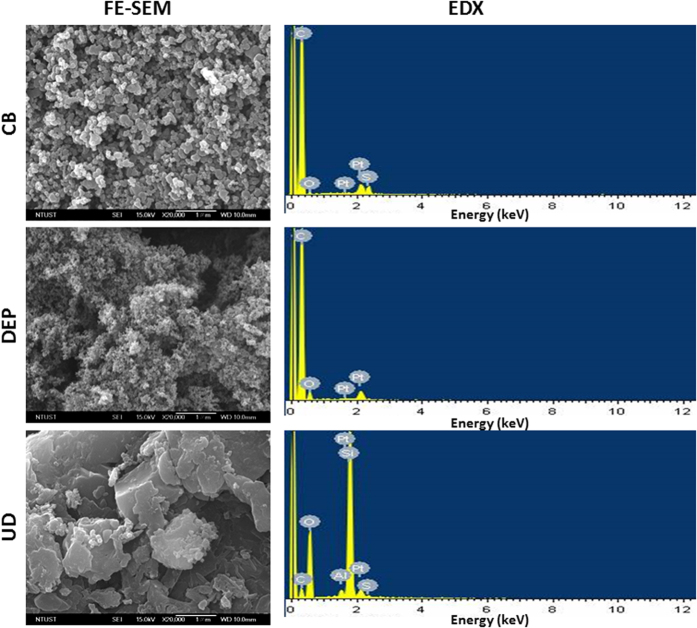
FE-SEM and EDX analyses of carbon black (CB), diesel exhaust particles (DEPs), and urban dust (UD). CB and DEP were spherical, and UD was amorphous in aggregates. UD was larger than CB and DEPs in size. Some elements were observed in these particles: C, O, and S in CB; C and O in DEPs; and C, O, Al, Si, and S in UD.

The SRM of DEPs (representing diesel-related traffic particles) and UD (representing ambient urban particles) were used to examine protein oxidation and degradation in A549 cells, whereas CB (nearly pure carbon element) was used in this study as a negative control. Spherical CB and DEP and amorphous UD aggregates were the dominant form observed by FE-SEM (Fig. 1). The size of DEPs was significantly smaller than those of CB and UD according to FE-SEM images, whereas UD had the largest size among these particles. For the EDX analysis, only a few elements were observed in these particles: C, O, and S in CB; C and O in DEPs; and C, O, Al, Si, and S in UD.

### Cell survival and proliferation

**Figure 2:**
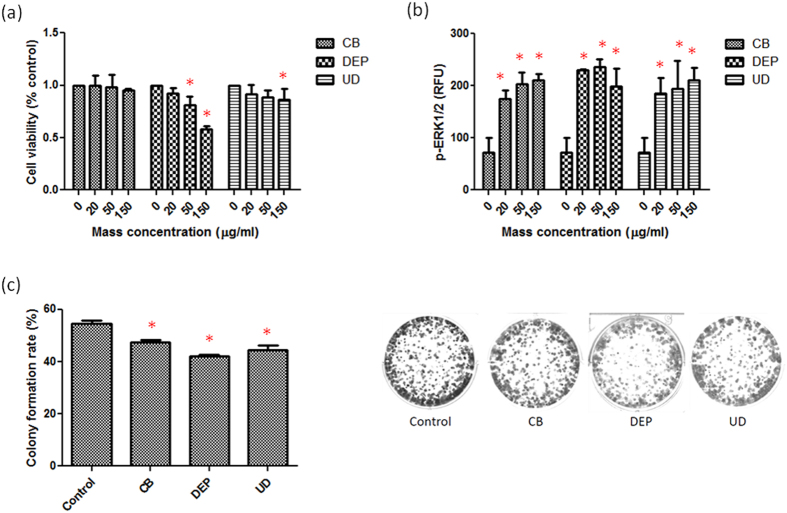
Effects of carbon black (CB), diesel exhaust particles (DEPs), and urban dust (UD) on the survival and proliferation of A549 cells. (**a**) Cell viability was determined in response to CB, DEPs, and UD at 0, 20, 50, and 150 μg/ml for 24 h. (**b**) ERK1/2 phosphorylation (p-ERK1/2) was determined in response to CB, DEPs, and UD at 0, 20, 50, and 150 μg/ml for 24 h. (**c**) Colony formation was analyzed in response to CB, DEPs, and UD at 20 μg/ml. Cell survival and proliferation were significantly reduced by DEPs and UD via ERK1/2 phosphorylation. *p < 0.05.

To investigate the particle toxicity among CB, DEPs, and UD, cell viability, p-ERK1/2, and colony formation were determined in A549 cells. First, we found that a significant reduction of cell viability occurred with DEPs at 50 and 150 μg/ml and UD at 150 μg/ml (*p* < 0.05) (Fig. 2a). There was no significant change in cell viability in response to CB exposure at 20~150 μg/ml. Figure 2b shows that levels of p-ERK1/2 significant increased after exposure to CB, DEPs, and UD at 20, 50, and 150 μg/ml (*p* < 0.05), particularly DEPs. The results show that these particles activated ERK phosphorylation with 24 h of exposure, leading to apoptosis. The survival and proliferation of A549 cells after 20 μg/ml CB, DEP, and UD exposure were further assessed using a colony formation assay. As shown in Fig. 1c, the three particles significantly affected growth rates of A549 cells (*p* < 0.05). Notably, the growth rate of DEP-treated A549 cells was slower than those treated with CB and UD.

### Protein oxidation and repair

**Figure 3:**
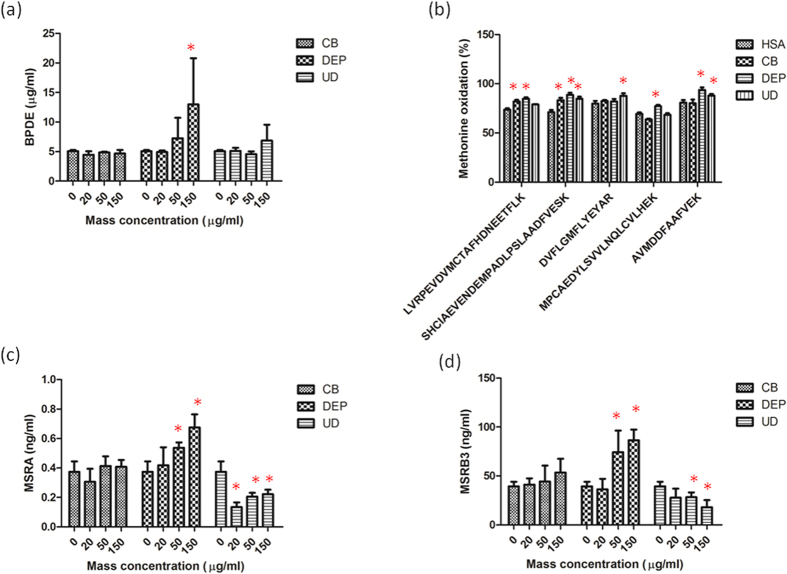
Effects of carbon black (CB), diesel exhaust particles (DEPs), and urban dust (UD) on protein oxidation. (**a**) Benzo(a)pyrene diolepoxide (BPDE) oxidation was determined after exposure of A549 cells to CB, DEPs, and UD at 0, 20, 50, and 150 μg/ml for 24 h. (**b**) Methionine oxidation (MetO) was examined. in human serum albumin (HSA) by CB, DEPs, and UD at 150 μg/ml for 24 h (**c**) MSRA expression was determined after exposure of A549 cells to CB, DEPs, and UD at 0, 20, 50, and 150 μg/ml for 24 h. (**d**) Methionine sulfoxide reductase B3 (MSRB3) expression was determined after exposure of A549 cells to CB, DEPs, and UD at 0, 20, 50, and 150 μg/ml for 24 h. The BPDE protein was oxidized by DEPs. MetO in five peptides of HSA caused by CB, DEPs and UD was identified. The protein repair mechanism that converts MetO back to methionine by MSRA and MSRB3 was activated by DEPs and inhibited by UD. *p < 0.05.

Protein oxidation caused by CB, DEPs, and UD was first assessed by BPDE protein oxidation (Fig. 3a). The BPDE protein was significantly oxidized by DEPs at 150 μg/ml in A549 cells (*p* < 0.05), whereas there was no significant alteration in BPDE protein oxidation caused by CB or UD. Methionine oxidation caused by the particles was examined because methionine can reduce BPDE oxidation. Significant oxidation of five peptides was observed for CB (82% for LVRPEVDVMCTAFHDNEETFLK and 83% for SHCIAEVENDEMPADLPSLAADFVESK), DEPs (85% for LVRPEVDVMCTAFHDNEETFLK, 89% for SHCIAEVENDEMPADLPSLAADFVESK, 77% for MPCAEDYLSVVLNQLCVLHEK, and 94% for AVMDDFAAFVEK), and UD (85% for SHCIAEVENDEMPADLPSLAADFVESK, 88% for DVFLGMFLYEYAR, and 88% for AVMDDFAAFVEK) compared to the HSA control (*p* < 0.05). Distinct effects of CB, DEPs, and UD on MSRA and MSRB were observed in A549 cells (Fig. 3c,d). MSRA and MSRB levels significantly increased with DEP exposure in a dose-dependent manner (*p* < 0.05), but UD significantly reduced MSRA and MSRB levels after exposure (p < 0.05). Exposure of A549 cells to CB had no significant effects on MSRA or MSRB3 alterations. We observed that activation of MSRA and MSRB production occurred with DEPs, whereas MSRA and MSRB3 levels were suppressed by UD. These results indicated that DEPs and UD had distinct effects on the protein repair mechanism of converting MetO back to methionine by MSRA and MSRB3.

### Protein degradation

**Figure 4:**
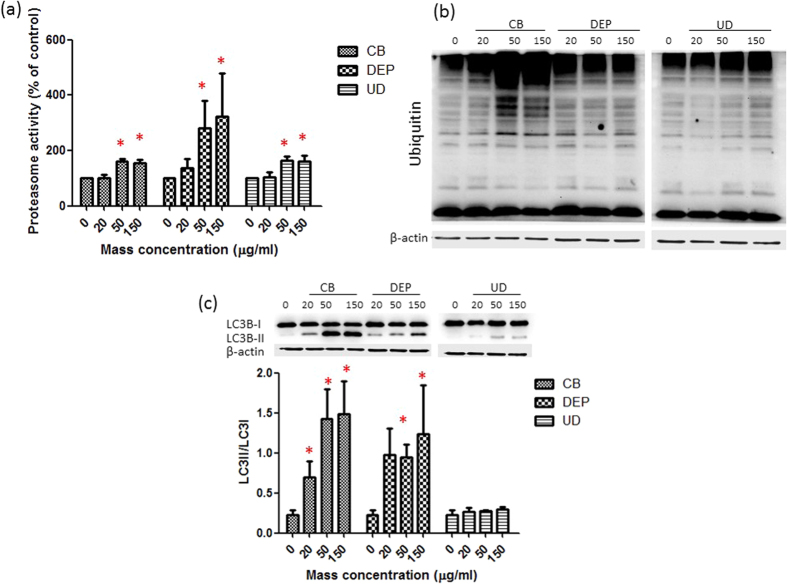
Effects of carbon black (CB), diesel exhaust particles (DEPs), and urban dust (UD) on protein degradation. (**a**) Proteasome activity was determined after A549 cells were exposed to CB, DEPs, and UD at 0, 20, 50, and 150 μg/ml for 24 h. (**b**) Expression of ubiquitin was determined after A549 cells were exposed to CB, DEPs, and UD at 0, 20, 50, and 150 μg/ml for 24 h. (**c**) LC3B II expression (adjusted by LC3B I) was determined after exposure of A549 cells to CB, DEPs, and UD at 0, 20, 50, and 150 μg/ml for 24 h. Proteasome and autophagy activation was induced by CB with ubiquitin accumulation, whereas proteasome and autophagy activation was induced by DEPs without ubiquitin accumulation. CB-induced protein degradation was via an ubiquitin-dependent autophagy pathway, whereas DEP-induced protein degradation was via an ubiquitin-independent autophagy pathway. *p < 0.05.

To determine if protein degradation occurred after exposure to these particles, proteasomes and ubiquitin were first investigated. Figure 4a shows that proteasome activity significantly increased after exposure to 50 and 150 μg/ml CB, DEPs, and UD (*p* < 0.05), particularly DEPs. Interestingly, we observed that CB significantly increased ubiquitin expression compared to the control (Fig. 4b), whereas UD did not have a significant effect on ubiquitin expression. Next, we examined the expression of LC3B II (after controlling LC3B I) after exposure to CB, DEPs, and UD (Fig. 4c). We observed that LC3B I was significantly converted to LC3B II by CB at 50 and 150 μg/ml (*p* < 0.05), whereas LC3B I to II conversion significantly occurred by DEP exposure at 150 μg/ml (*p* < 0.05). There was no significant effect of UD on LC3B conversion.

## Discussion

Protein oxidation in the lung environment has been linked to several lung diseases such as chronic obstructive pulmonary disease (COPD), which is considered to play an important role in regulating the pathophysiology of lung diseases^19^,^20^. However, proteotoxicity induced by particulate air pollution remains unclear. The present study discovered differences in protein oxidation and degradation in A549 cells exposed to CB, DEPs, and UD. Five major findings are reported in the present study: (1) cell survival and proliferation were significantly reduced by DEPs and UD; (2) methionine oxidation (MetO) occurred after CB, DEP, and UD exposure; (3) the protein repair mechanism that converts MetO back to methionine by MSRA and MSRB3 was activated by DEPs and inhibited by UD; (4) proteasome and autophagy activation was induced by CB with ubiquitin accumulation, whereas proteasome and autophagy activation was induced by DEPs without ubiquitin accumulation; and (5) CB-induced protein degradation was via a ubiquitin-dependent autophagy pathway, whereas DEP-induced protein degradation was via a ubiquitin-independent autophagy pathway.

Ambient PM presents unique and often distinct physicochemical properties, as the physiochemical properties change from generation to secondary formation/interactions, which contribute to particle toxicity. In the present study, three types of PM were used to determine the potency of protein oxidation and degradation, and these were also used for a mechanical study^21^. DEPs, SRM chemical-rich particles, were generated by a heavy-duty forklift diesel engine^22^, which represented diesel-related traffic pollution. UD, SRM atmospheric chemical-rich particles, was collected from an urban area^23^. CB, a carbon-core control particle, was produced by the thermal decomposition of hydrocarbons^24^. We observed that CB and DEPs were spherical when aggregated, but UD was amorphous with minor inorganics when aggregated. With different physicochemical characteristics of the three particles, we expected to observe distinct proteotoxicities induced by these particles.

Alterations of cell survival and proliferation caused by CB, DEPs, and UD were first examined. We observed that DEPs had higher particle toxicity than CB or UD, which is consistent with a previous report^25^. However, CB had no significant effects on cell viability after exposure. We further showed that activation of ERK phosphorylation occurred after exposure to the three particles. ERK controls various aspects of cell physiology, such as apoptosis and proliferation^26^, and is also linked to the regulation of protein degradation^27^,^28^. For example, ERK signaling regulates expressions of autophagic and lysosomal genes^27^. Our results indicate that DEPs are more toxic than CB and UD in activating cell death.

ROS are reactive molecular species with an unpaired electron in their outer orbit^29^, so they can easily extract a second electron from a neighboring molecule. Previous reports showed that BPDE is formed due to protein adduct caused by benzo[a]pyrene^30^ and metals^31^. Our results showed that significant BPDE protein damage occurred by DEP exposure of A549 cells. We then determined levels of methionine oxidation in HSA, because methionine has chemoprotective effects on reducing levels of the BPDE adduct^32^. Our results showed that methionine was significantly oxidized by the three particles. Interestingly, the methionine repair system, i.e., MSRA and MSRB3, displayed distinct responses after CB (no effect), DEP (increase), and UD (decrease) exposure. ROS-induced methionine to methionine oxidation can be reversed by MSRA which reduces *S*-MetO and MSRB which reduces *R*-MetO^33^, thus maintaining protein functions and reducing the accumulation of damaged proteins in cells. In the present study, we observed that the protein repair mechanism of converting oxidized methionine back to methionine by MSRA and MSRB3 was activated by DEPs, which could mitigate oxidized protein accumulation. But the MSR repair system was dysregulated by UD, therefore leading to a cellular MSRA and MSRB3 deficiency. UD-induced dysfunction of the MSR system may cause oxidized proteins to accumulate in cells. Different self-repair mechanisms of protein oxidation induced by the particles were observed in our study, implying that particles could have different abilities to regulate protein degradation.

Next, we investigated regulation of protein degradation pathways when an overload of oxidized protein accumulated in cells caused by the particles. Biologically, intracellular proteins are mainly degraded by the ubiquitin-proteasome pathway^34^. Cigarette smoke increased accumulation of polyubiquitinated proteins in soluble and insoluble protein fractions in lungs of mice and human epithelial cells, suggesting impairment of proteasome activity in the lungs^35^. Proteasomes are able to selectively recognize and degrade mildly oxidized proteins in the cytosol, nuclei and endoplasmic reticula, thereby minimizing the proteotoxicity caused by those particles. Oxidized proteins are actively recognized and degraded by the 20S proteasome complex, but the 26S proteasome complex is less effective^36^. Ubiquitin tagging of proteins is recognized by the 26S proteasome, which degrades ubiquitinated proteins to small peptides^37^. In the present study, proteasome activity was activated by exposure of A549 cells to CB, DEPs, and UD; however, distinct expressions of ubiquitin were observed. Ubiquitin is a link chain of a polypeptide co-factor that makes proteins for degradation^36^. Therefore, the 26S proteasome may have different roles in response to CB, DEPs, and UD. Furthermore, there is the possibility that the three particles have different regulatory mechanisms in E1 (ubiquitin-activating enzyme), E2s (ubiquitin-carrier or conjugating proteins), and E3 (ubiquitin-protein ligase). Our results suggest that DEPs and UD may disrupt the 26S proteasome complex or E1, E2, and E3; however, the possible association should be investigated in future work.

Upon encountering oxidative stress, proteins are extensively oxidized by ROS, and these oxidized proteins are degraded by proteasome and autophagic proteolytic systems for bulk degradation. Deng and colleagues showed that ambient PM regulated the autophagy-related proteins, Atg5 and Beclin 1, which are associated with particle-driven oxidative stress^17^. Moschini and colleagues noted that autophagy was involved in the response of A549 cells exposed to nanoscale metal-based particles^38^. Furthermore, Chen and colleagues suggested that autophagy is an important mechanism underlying inflammation and mucus hyperproduction induced by nanoscale particles in the airway epithelium^39^. In the present study, we found that CB and DEPs significantly increased LC3B II expression; however, there was no effect on LC3B II conversion by UD. The difference may have resulted from the distinct physicochemical features of these particles, suggesting that the expression of autophagy may be regulated of particle byproducts.

Notably, taken together with the expression of ubiquitin, we observed that CB exposure was associated with ubiquitin-dependent autophagy, whereas DEPs were associated with ubiquitin-independent autophagy even at the lowest concentration. The effects of lower dose of DEP could be represented in a real-world exposure. Oxidized proteins are tagged with ubiquitin signals that are subsequently sensed by ubiquitin binding domain-containing receptors to deliver them to proteasomes or autophagosomes during autophagy^40^. Selectivity of autophagy is controlled by specialized autophagy receptors, that physically link defined cellular material with the autophagy compartment by interacting simultaneously with cargo and Atg8- or LC3/GABARAP-like proteins on autophagosomes^41^. In ubiquitin-dependent selective autophagy, autophagy receptors simultaneously recognize ubiquitin chains attached to intracellular cargo (e.g., CB) via ubiquitin-binding domains, thereby linking targeted material to the autophagosomal membrane^42^. On the other hand, with ubiquitin-independent autophagy, autophagy receptors directly bind to intracellular cargo (e.g., mitochondria), in some cases via transmembrane domains. Autophagy receptors tend to cluster their cargo through specialized oligomerization domains^42^. In addition to ubiquitin-mediated selective autophagy, cellular cargo can be delivered for autophagy independently of ubiquitin. Based on recent reports, selective autophagy has emerged as an important mechanism in pulmonary disease. Exposure to cigarette smoke was linked to regulation of a selective autophagy pathway in cilia length^43^. Finally, our results suggest a possible autophagy pathway regulated by particle byproducts. More evidence should be provided to confirm and clarify the role of selective autophagy and the related mechanisms in air pollution.

## Conclusions

**Figure 5:**
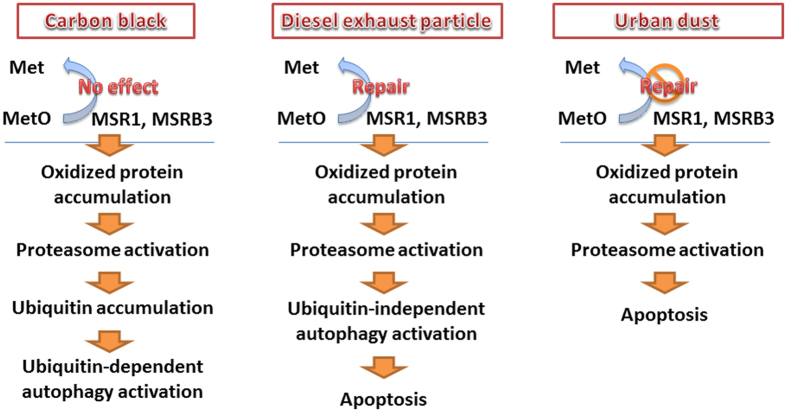
Illustration of the hypothetical pathways of protein oxidation and degradation in response to exposure to carbon black (CB), diesel exhaust particles (DEPs), and urban dust (UD). First, the methionine sulfoxide reductase (MSR) system worked well to repair oxidized proteins caused by DEPs; however, the MSR system was abnormal with UD exposure and showed no effects with CB exposure. Proteasomes were then activated by the three particles after an accumulation of oxidized proteins, but only CB increased ubiquitin accumulation in A549 cells. Next, selective autophagy, i.e., ubiquitin-dependent autophagy for CB and ubiquitin-independent autophagy for DEPs, may be regulated by particle byproducts. Finally, apoptosis was activated by DEPs and UD.

This study showed a proteotoxic effect, of regulation by the MSR repair system, the proteasome-ubiquitin pathway, and selective autophagy, after exposure of A549 cells to CB, DEPs, and UD. Based on our findings, we propose the distinct proteotoxicity of CB, DEPs, and UD. The hypothetical pathways of protein oxidation and degradation in response to exposure to CB, DEP, and UD are shown in Fig. 5. First, the MSR system can work well to repair oxidized proteins caused by DEPs; however, the MSR system was abnormal after exposure to UD, but no effects occurred with CB exposure. Proteasomes were activated by the three particles after the accumulation of oxidized proteins, but only CB increased ubiquitin accumulation in A549 cells. Next, selective autophagy, i.e., ubiquitin-dependent autophagy for CB and ubiquitin-independent autophagy for DEPs, may be mediated by particle byproducts. Finally, apoptosis was activated by DEPs and UD. Different responses in proteotoxicity caused by CB, DEPs, and UD may depend on the physicochemistry of the particles; therefore, it is important to understand the physicochemical effects of ambient particles on protein oxidation and degradation in initiating pulmonary diseases. Determination of protein oxidation and degradation in normal lung cells, such as human bronchial epithelium cells, is required for the comparison with the current results. Also, mimicking lung environment such as air-liquid-interface (ALI) system would be helpful to determine the effects of particles in physiologically imitating conditions. Our findings provide additional evidence, for the first time, that distinct proteotoxicities occurred with different types of particulate air pollution, which may be associated with underlying mechanisms for developing environmental lung diseases.

## Materials and Methods

### Particles and reagent sources

Diesel exhaust particles (DEPs) and urban dust (UD) as Standard Reference Materials (SRM) 2975 and 1649b, respectively, were obtained from the National Institute of Standards and Technology (NIST, Gaithersburg, USA). Carbon black (CB), with an average diameter of 65 nm (Monarch 120; Cabot, UK), was selected as a control particle. All of the other reagents were obtained from Sigma (St. Louis, MO, USA) if a source is not explicitly stated.

### Field emission-scanning electron microscopy (FE-SEM) and energy-dispersive x-ray (EDX) microanalysis

Inspect™ FE-SEM (JEOL 2100, Jeol, Japan) and an EDX microanalysis were used to investigate morphological and elemental features of the particles. Preparation of the FE-SEM samples was previously described^44^. Briefly, particles adhered to 12-mm carbon sticky tabs, which were fixed on 13-mm aluminum SEM stubs. Samples were coated with platinum to an average thickness of 10 nm using a sputter-coater and then imaged. FE-SEM was operated at an accelerating voltage of 15 kV and a 2.5-nm spot size. The EDX analysis was performed using the EDX Genesis Microanalysis System.

### Cell culture and treatment

Human lung epithelial A549 cells obtained from the American Type Culture Collection (ATCC, Manassas, VA, USA) were seeded in surface-treated, 96-well transwells (BD Biosciences, Oxford, UK) at a density of 10^5^ cells/ml and incubated for 24 h. Cells were cultured in RPMI medium containing 10% fetal bovine serum, penicillin, and streptomycin and were incubated at 37 °C with 95% humidity and 5% CO_2_. Particles were freshly prepared in serum-free RPMI medium at mass concentrations of 0, 20, 50, and 150 μg/ml. Cells were then incubated at 37 °C for 24 h in a humidified atmosphere with 5% CO_2_.

### Cell viability

A sulforhodamine B (SRB) colorimetric assay was used to examine cell viability according a previous method^45^. Briefly, cells were fixed with 10% (wt/vol) trichloroacetic acid and stained for 30 min. The protein-bound dye was dissolved in 10 mM of a Tris base solution after removing excess dye, and the OD was measured at 510 nm using a microplate reader. The cell viability is presented as a percentage (%) after adjusting for the control.

### Colony formation assay

The protocol of the colony formation assay was described previously^46^. Briefly, 500 cells were resuspended in RPMI medium and seeded in six-well surface-coated plates following a 24-h incubation. Cells were exposed to 20 μg/ml of particles for 5 days. Next, cells were washed and cultured for a further 9 days. Finally, cells were washed and fixed with 3.7% paraformaldehyde following staining with 0.05% crystal violet. The plates were stained with 0.5 mg/ml p-iodonitrotetrazolium violet. Colonies with a diameter of >1 mm were counted. Triplicate samples were used in the experiment. Images were then analyzed for the colony formation rate (%) using ImageJ software (vers. 1.49, Bethesda, MD, USA)^47^.

### Methionine oxidation

Methionine oxidation by particles was previously reported^10^,^48^. Briefly, a 1 mg/ml solution of recombinant human serum albumin (HSA) was prepared with sterile phosphate-buffered saline (PBS) and then vortexed with 150 μg/ml of particles for 2 h. Samples were diluted with 6.5 mM dithiothreitol and then alkylated using 10 mM iodoacetamide. A Q-Exactive Mass Spectrometer (MS) (Thermo Fisher Scientific, Bremen, Germany) equipped with an UltiMate 3000 RSLC system was used to determine the tryptic peptides after digestion. Liquid chromatography equipped with a C18 column (Acclaim PepMap RSLC, 75 μm × 150 mm, 2 μm, Dionex) was used to separate the peptides. Full MS scans were performed with an m/z range of 300~2000, and the ten most intense ions from the MS scan were subjected to fragmentation to yield MS/MS spectra. The raw data were processed into peak lists by Proteome Discoverer vers. 1.4 for Mascot database searches (http://www.matrixscience.com). The search parameters specified variable modification for deamidation (NQ), oxidation (M), and methylation (K) and a fixed modification for carbamidomethylation (C). The maximum mass tolerance was set to 10 ppm for precursor ions and 0.05 Da for fragmented ions. The degree of oxidation was calculated based on the peak area [(peak area of oxidized peptides/total peak area of peptides with and without oxidation) × 100%]. The Swiss-Pdb Viewer vers. 4.1.0 (Swiss Institute of Bioinformatics, Switzerland) was used to analyze the peptides oxidized in HSA oxidized by the particles^49^.

### Enzyme-linked immunosorbent assay (ELISA)

Cells were collected form the plates and stored at −20 °C overnight in 1x PBS after particle exposure. After two freeze-thaw cycles to break up cell membranes, cell lysates were centrifuged at 5000 x*g* and 2 °C. Supernatants were collected to determine phosphorylated extracellular signal-regulated kinase (ERK)1/2 (p-ERK1/2) (ERK1/2 pThr202/Tyr204 + Total PhosphoTracer ELISA Kit; Abcam, Cambridge, MA, USA), benzo(a)pyrene diolepoxide (BPDE) protein adduct (OxiSelect™, Cell Biolabs, San Diego, CA, USA), MSRA (Uscn Life Science, Wuhan, Hubei, China), and MSRB3 (Uscn Life Science) and proteasome activity (Abcam) using an ELISA according to the manufacturer’s instructions.

### Western blot analysis

Western blot analyses were performed as described previously^50^. Briefly, cells were cultured in 6-cm dishes for particle exposure. Proteins isolated from lysed cells were subjected to sodium dodecyl sulfate polyacrylamide gel electrophoresis (SDS-PAGE) and were electrotransferred onto polyvinylidene difluoride (PVDF) membranes (Millipore, Darmstadt, Germany). The primary antibodies for ubiquitin (1:1000), LC3B (1:1000), and β-catenin (1:1000) were obtained from Cell Signaling (Danvers, MA, US). Anti-mouse (1:5000) and anti-rabbit (1:2000) horseradish peroxidase (HRP)-conjugated secondary antibodies were obtained from Chemicon International (MA, USA). Blots were then blocked with 5% skim milk in PBST and were probed with primary antibodies overnight at 4 °C. An HRP-labeled secondary antibody was then incubated for 60 min at room temperature and washed with TBST. Enhanced chemiluminescence (ECL) Western blotting reagents were used, followed by imaging using the BioSpectrum Imaging System (UVP, Upland, CA, USA). Quantitative data were obtained using ImageJ software^47^.

### Statistical analysis

All experiments were performed in quintuplicate. Data are expressed as the mean ± standard deviation (SD). A one-way analysis of variance (ANOVA) with Tukey’s post-hoc test was used for comparisons among multiple values. Statistical analyses were performed using GraphPad vers. 5 for Windows. The level of significance was set to *p* < 0.05.

## Additional Information

**How to cite this article**: Lai, C.-H. *et al*. Protein oxidation and degradation caused by particulate matter. *Sci. Rep*. **6**, 33727; doi: 10.1038/srep33727 (2016).
